# Henke’s Atlas of Surgical Anatomy

**Published:** 1884-11

**Authors:** 


**Henke's Atlas of Surgical Anatomy.** A series of plates illustrating the application of anatomy to medicine and surgery. Translated and edited by W. A. RATHACKER, M. D., Pathologist to Cincinnati Hospital, Lecturer on Pathological Anatomy, Miami Medical College. Cincinnati: A. E. Wilde & Co. 1884.

This large and handsome volume contains eighty-one plates. Those who have the good fortune to possess the admirable "Atlas of Anatomy" published by this same house will not need to be told that the engravings are excellent and the typography and press-work all that could be desired. This work is, to many, even more valuable than the atlas referred to, in that it shows us, at a glance, the anatomy of the human body as we view it in relation to surgical operations, medical diagnosis, post mortem examinations, etc. It is a work that will prove of the greatest value to the student, the surgeon and the physician. To the student, not to take the place of dissection, but to fix the application of its lessons in his mind. It shows him the structures grouped together just as he exposes them. To the surgeon, who may have become "rusty" concerning some points in anatomy, the parts are pictured in the order in which he divides them with his knife. To the physician, it furnishes a ready means of keeping fresh in his mind the lessons of his student days. The price at which it is offered is remarkably low ($10). The work ought to, and will, undoubtedly, meet with a large sale.

